# Taxonomy and Phylogenetic Research on *Ralstonia solanacearum* Species Complex: A Complex Pathogen with Extraordinary Economic Consequences

**DOI:** 10.3390/pathogens9110886

**Published:** 2020-10-25

**Authors:** Sujan Paudel, Shefali Dobhal, Anne M. Alvarez, Mohammad Arif

**Affiliations:** Department of Plant and Environmental Protection Sciences, University of Hawaii at Manoa, Honolulu, HI 96822, USA; sujanpau@hawaii.edu (S.P.); shefali@hawaii.edu (S.D.)

**Keywords:** *Ralstonia solanacearum* species complex, taxonomy, phylogenomic, plant bacteria, tomato wilt, bacterial wilt, brown rot of potato, Granville Wilt of tobacco, moko disease of banana, Bugtok disease

## Abstract

The bacterial wilt pathogen, first known as *Bacillus solanacearum*, has undergone numerous taxonomic changes since its first description in 1896. The history and significance of this pathogen is covered in this review with an emphasis on the advances in technology that were used to support each reclassification that finally led to the current separation of *Ralstonia solanacearum* into three genomic species. Frequent name changes occurred as methodology transitioned from phenotypic, biochemical, and molecular studies, to genomics and functional genomics. The diversity, wide host range, and geographical distribution of the bacterial wilt pathogen resulted in its division into three species as genomic analyses elucidated phylogenetic relationships among strains. Current advances in phylogenetics and functional genomics now open new avenues for research into epidemiology and control of the devastating bacterial wilt disease.

## 1. Introduction

The global impact of plant pathogenic bacteria on plants is significant, the greatest impact occurring in warm and humid regions of the world [1]. Among the economically important plant pathogenic bacterial species, the beta proteobacterium *Ralstonia solanacearum* (synonyms: *Bacillus solanacearum*, *Bacterium solanacearum*, *Phytomonas solanacearum*, *Xanthomonas solanacearum*, *Pseudomonas solanacearum, Burkholderia solanacearum*) is of particular interest to the scientific community because of its global distribution, wide host range (over 250 plant hosts in 54 plant families), aggressiveness under diverse environmental conditions [2,3] and its remarkable ability to retain pathogenicity genes in the absence of a host plant [4,5]. Commonly known as bacterial wilt, this bacterial disease has several other names such as brown rot of potato, Granville Wilt of tobacco, moko disease of banana, Bugtok disease, spewy eye, and other descriptive names. Plants affected by *R. solanacearum* include, but are not limited to, staple crops (potato), fruit crops (banana, tomato), oilseed crops (sunflower, groundnut), spice crops, fodder, flowers, forest trees (ironwood and eucalyptus), weeds and many ornamentals. *R. solanacearum* was listed as the second most destructive among plant pathogenic bacteria and has serious economic consequences [6]. Crop losses due to this pathogen worldwide are extremely difficult to evaluate due to irregularity in data collection; nevertheless, Elphinstone (2005) reported annual estimated losses estimated at one billion USD [7].

Bacteria in the *Ralstonia solanacearum* species complex (RSSC) have been associated with new hosts worldwide, posing a serious threat to agricultural production. The continual emergence of new RSSC hosts is linked to the ever expanding phylotype I species group with long range migration and high recombination potential [8]. The cold-tolerant, aggressive brown-rot strains are known to affect the potato industry in all the continents, particularly in the highlands of tropical, subtropical, and temperate regions of the world. The pathogen is reported to affect 3.75 million acres (15,175 sq. km) of potato production in 80 countries worldwide [9]. Likewise, the monetary losses to the agricultural economy of the developed world due to bacterial wilt can be immense due to the high value—for instance, the potato and tomato industries in the US alone are valued at $3.7 billion [10] and $1.67 billion [11], respectively. Furthermore, the ease of dissemination of this pathogen through irrigation water, farm tools and crop debris pose a serious threat to subsistence agriculture in developing countries [12]. Saprophytic survival and efficient spread through routine agricultural operations makes the control of this pathogen extremely difficult. Thus, the pathogen has stimulated remarkable social, economic and research interest.

**Figure 1:**
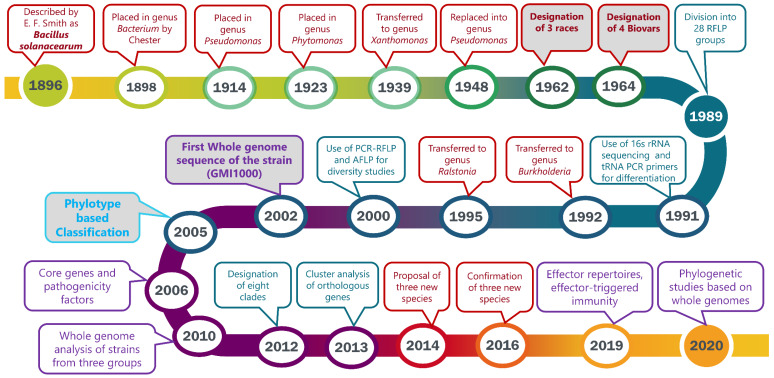
Timeline highlighting the major events based on taxonomic and phylogenetic studies of the bacterial wilt pathogen now in the *Ralstonia solanacearum* species complex (RSSC). Events in red boxes with red fonts indicate major changes in taxonomy and nomenclature. Blue boxes with blue fonts indicate milestones in the DNA-based analyses of RSSC. Purple boxes with purple fonts represent genomic advancements in understanding the diversity of multiple strains of RSSC. Bold labels with grey backgrounds indicate landmark taxonomic and genomic events. Landmark publications associated with each date are provided in the text associated with the date.

The taxonomy and nomenclature of the “bacterial wilt pathogen” changed frequently with the evolution of new technologies. First named *Bacillus solanacearum* [13], the pathogen was reclassified multiple times before it was transferred to the current genus, *Ralstonia*. A historical review will emphasize the advances in technology that enabled increasingly greater precision in bacterial classification. Phylogenetic and evolutionary studies have been widely applied to address the significant heterogeneity among strains. The discovery of new strains from different geographical habitats has prompted further reclassification based on genomic analyses. The major events in the taxonomic history of this organism over the past 124 years are shown in the timeline (Figure 1). Genome organization, pathogenicity-related effectors, virulence pathways, and genome-based phylogenetic relationships have increased our understanding of this heterogeneous pathogen.

## 2. 1896–1963: The Discovery and Early Classification

Bacterial wilt symptoms were observed on multiple hosts in 1890 but little proof of pathogenicity was provided, and no descriptions of the causal organism were included in early studies. The wilt disease was described on potato in 1890 by T.J. Burrill, the first to isolate and re-inoculate the pathogen [14,15]. Bacterial wilt of tomato was observed in Mississippi 1891 by Byron D. Halsted, who attributed the disease to the same causal agent as he previously observed on cucumber and cantaloupe [16]. In 1895, E. F. Smith isolated the bacterial wilt pathogen from both tomato and potato and demonstrated that it was different from the bacterial pathogen affecting cucumber [17,18]. Smith’s studies of the biology, pathogenicity, and the nature of symptoms expressed on multiple hosts led him to name a new bacterial species—*Bacillus solanacearum* [13]. Chester (1898) placed the organism into the genus *Bacterium* based on key bacteriological features including polar, rather than peritrichous, flagella [19]. The bacterial wilt pathogen was later confirmed to have a single polar flagellum, and Chester’s classification was accepted by Smith [20]. The first disease descriptions were made in the southern states of the US [14,16,17]. Later, bacterial wilt of banana and other crops was described in Central America, Philippines, and Indonesia, and many other tropical and subtropical countries. Several bacterial wilt diseases were attributed to *Bacillus* sp., including “moko” (or slime disease) of banana, which was ascribed to *Bacillus musae* [21]. Smith proposed that these bacterial wilt pathogens be designated either *Bacterium solanacearum* or *Pseudomonas solanacearum*, based on Migula’s description of the genus *Pseudomonas* [22]. Subsequently, this pathogen underwent further reclassifications to *Phytomonas solanacearum* [23] and *Xanthomonas solanacearum* [24] based on comprehensive studies of bacteriological characteristics, including the number and position of flagella. Following additional studies of multiple physical, nutritional, and biochemical properties by different investigators, Breed, et al., Savulescu, and Dowson were in consensus to replace the organism back into *Pseudomonas* [17,23,25,26,27].

## 3. 1964: Sub-Classification of *Pseudomonas solanacearum* into Races and Biovars

In 1962, Buddenhagen, Sequeira, and Kelman characterized multiple strains of *Pseudomonas solanacearum*—isolated from Latin America and the United States—based on pathotype, colony type, serotype, biochemical type, lysotype, and bacteriocinotype and grouped the strains into three broad categories known as races [28]. Strains in race 1 induced symptoms on a broad host range including solanaceous crops and diploid banana; race 2 was associated with moko (slime) disease of triploid banana, *Heliconia* or both, while race 3 was primarily pathogenic to potato and tomato [29]. These race designations were principally based on pathogenicity to various hosts and phenotypic characteristics of the bacterial strains, whereas the concept of *race* for pathogenic fungi was defined by the ability of a pathogen to infect a specific host *variety* or *cultivar* (for example, races of *Puccinia graminis* were pathogenic on specific varieties of wheat). In contrast, the term *race* as then applied to *P. solanacearum* indicated pathotype (specificity to host genera and/or species) in conformance to the subsequent use of the term *pathovar* [30]. Buddenhagen and Kelman (1964) classified their strains into four biochemical types (or biovars) based on the biochemical system devised by Hayward (1964) [29,31]. The biotype designation was based on the ability of a strain to oxidize three disaccharides (sucrose, maltose, lactose) and three hexose alcohols (mannitol, sorbitol, dulcitol) (Table 1). Hayward found that 95 strains isolated from potato in different geographical locations worldwide were all biotype 2. However, potato also was affected by the broad host range strains of *P. solanacearum* in race 1, and except for the observation that most race 3 potato strains were also classified as biotype 2, no other direct relationship between race and biotype was found [29,31].

In a study conducted on 29 strains isolated from 14 cultivated and wild host plants in China He et al., reported that strains affecting sweet potato, olive, casuarina, and several other hosts were in biotypes 3 and 4 [32]. However, strains from mulberry showed unusual characteristics with respect to pathogenicity and biochemical tests; thus, a new group—race 4, biotype 5—was proposed to include mulberry strains [32]. However, Aragaki and Quinon had previously described a novel *P. solanacearum* race from ginger, which was further described as biovar 4 by Pegg and Moffet [33,34]. The mulberry strain was later renamed race 5, biotype 5 [35].

The use of a race/biovar (biotype) system of classification based on host range and selected biochemical properties used for differentiation of strains within *P. solanacearum* [28,31,32] was informal and did not follow the Code of Nomenclature as no specific characteristics were discovered that permitted a formal classification system. The race and biovar system, while useful, failed to unravel the details about the origin and evolution of *P. solanacearum*. While some strains only affected the host grown in a specific geographical region, notable diversity also existed among strains isolated from the same host. This caused a problem in classification of the strains based on the host of origin [36]. Hayward suggested that the differences in nutritional patterns and geographical sites of origin be used to formally classify the strains and predicted that the species would be divided into subspecies in the imminent future [2].

## 4. 1989: Classification into Two Divisions Based on RFLP Patterns

Restriction fragment length polymorphism (RFLP) analysis was used by Cook, et al. to develop a classification system for *P. solanacearum* species based on 62 strains [36]. Differences in locations of unique restriction enzyme sites were observed using Southern Blots with cloned DNA fragments, which were used as probes that specified virulence and the hypersensitive response. Twenty-eight unique groups or multi-locus genotypes (MLGs) were discovered based on RFLP patterns and the calculation of similarity coefficients resulted in the placement of the 28 MLGs into two divisions. Division I comprised MLGs 8 to 23 and included all members of race 1 biovars 3 and 4 and 5. Division II consisted of race 1 biovar 1 strains, and all members of races 2 and 3. Division II comprised MLGs 1–7 and 24–28 and formed three subdivisions: (i) MLGs 1–7 consisted of race 1 biovar 1 strains from various hosts; (ii) MLGs 24, 25 and 28 were race 2 biovar 1 strains from banana, plantain, and heliconia; (iii) MLGs 26 and 27 included race 3 biovar 2 strains from potato. Several probes failed to distinguish between some strains in Division I, indicating a close relationship among these strains. Race 1 biovar 1 potato strains were isolated in Kenya, Costa Rica, and Australia whereas race 3 biovar 2 potato strains were isolated in Israel, Colombia, Sri Lanka, and Australia. The two major divisions showed a loose relationship between the strains and their overall geographical origins and, to a lesser extent, their hosts of origin. The probes used for this classification of strains specified virulence and hypersensitivity but Cook et al. found no significant correlations between host range and MLG group based on RFLP analysis [36]. Nevertheless, the 63 strains were clearly separated into two major divisions, and Cook et al., suggested that the groups may have evolved separately with respect to their geographical origins [36].

## 5. General Taxonomic Revision of the Genus *Pseudomonas*

While plant bacteriologists continued to depend on the race/biovar system of classifying the bacterial wilt pathogen, the “competition” method of DNA hybridization had been employed to determine the relatedness of strains in the genus *Pseudomonas* and the evolutionary history and relationships between many *Pseudomonas* species were analyzed using DNA–DNA hybridizations in vitro [37]. A high degree of competition reflected a close relationship between strains. Using DNA from two *P. solanacearum* strains, 769 and 776, as references and DNA from other *Pseudomonas* species as the competitor, no homology was observed between *P. solanacearum* and other species. However, an unusual DNA homology was reported between *P. solanacearum* strains 769 and 776, and *P. cepacia* strain 382, though they did not show similar phenotypic characteristics. The homology was re-evaluated using a direct DNA binding experiment and negligible homology was observed between the species [37].

Genome coding for ribosomal ribonucleic acid (rRNA) was highly conserved and appeared to be less variable than DNA [38,39]. The rRNA–DNA competition percentage was higher compared to the DNA–DNA competition percentage using the same reference and competitor strains for both hybridizations. Thus, Palleroni et al. used rRNA–DNA hybridization assays to compare species of *Pseudomonas* [40]. Five groups were established based on the rRNA–DNA homologies. *Pseudomonas solanacearum* was included in homology group II along with *P. pickettii, P. marginata* (*=P. gladioli*) *P. cepacia, P. pseudomallei, P. mallei,* and *P. caryophylli*. The intragroup competition percentage was higher compared to the intergroup percentage, indicating that members within the group were related.

Eight representative strains from five members of *Pseudomonas* homology group II were sequenced and rDNAs were amplified to determine their phylogenetic relationships [41]. Their dendrogram showed that *P*. *cepacia*, *P*. *andropogonis*, *P*. *caryophylli*, *P*. *gladioli* pv. *gladioli* were grouped into one cluster with 94.2% sequence similarity to each other. Likewise, *P. solanacearum* and *P*. *pickettii* were grouped with *Alcaligenes eutrophus* at 95.3% and 92.8% similarity, respectively, and high genetic homology was obtained between *P*. *pickettii* and *P*. *solanacearum*. Clustering of strains into two distinct groups was consistent with findings of the earlier studies [37,42,43,44]. Genomic diversity was also reflected in low DNA–DNA hybridization values (<70%) among reference strains [37,45].

## 6. 1992: Determination of *Pseudomonas solanacearum* Subgroups Using PCR Amplification and t-RNA Consensus Primers

Two t-RNA consensus primers, T3A and T5A, designed by Welch and McClelland were used to amplify a set of DNA fragments in 112 *P. solanacearum* strains using PCR, and after fingerprint analysis, three groups emerged [46,47]. Type 1 corresponded quite well to *P. solanacearum* strains in Division II described by Cook and Type 2 strains were represented by strains which cause Blood Disease of Banana (BDB) described by Eden-Green and Sastraatmadja; Type 3 strains included biovars 3, 4, and 5 and corresponded to Division I described by Cook et al. [36,48]. The unusual strains of *Pseudomonas syzygii* sp. nov., which cause Sumatra disease of cloves (*Syzygium aromaticum*) [49,50], were clearly distinct from other *P. solanacearum* strains in Types 1 and 3 and in some publications were considered type 4. Clove strain B9043 was biovar 1 and clove strain R142 was biovar 2, an unusual finding as biovar 2 was almost exclusively associated with potato race 3 strains. Additional comparisons of representative strains from international collections were needed before the taxonomy of *P*. *solanacearum* could be clarified.

Species-specific 16s rRNA genes were used as targets to identify strains at the species level [51], and PCR primers were developed to distinguish P. *solanacearum*, *P*. *pickettii* and P. syzygii [51]. A close relationship between *P. solanacearum*, *P*. *syzygii* and the BDB strains was confirmed but required further molecular studies to resolve their differences [51]. The nucleotide sequence and predicted structure confirmed the inclusion of *P. solanacearum, P. syzygii, P. pickettii* along with BDB in the beta subdivision of purple bacteria.

## 7. 1992: Transfer of *Pseudomonas* Homology Group II into the New Genus *Burkholderia*

Seven species in the *Pseudomonas* homology group II (*P. cepacia*, *P. mallei*, *P. pseudomallei*, *P. pickettii*, *P. solanacearum, P. gladioli,* and *P. caryophylii*) were reclassified based on their phenotypic characteristics, cellular lipid and fatty acid composition, DNA–DNA homology values and 16s rRNA sequencing [52]. Five strains in homology group II, selected from *P. cepacia*, *P. mallei*, *P. pseudomallei*, *P. pickettii*, and *P. solanacearum*, were used as type strains and two strains of *P. gladioli* and *P. caryophylii* were used as reference strains. Strain EY274 of *P. aeruginosa* was used for taxonomic comparison. The polar lipid and fatty acid composition showed that these *Pseudomonas* strains diverged widely from the existing genus *Pseudomonas*, leading Yabuuchi et al. to propose a new genus *Burkholderia* [52]. The DNA–DNA homology comparisons and the phylogenetic analyses strengthened the case for transfer of the group into the new genus [52].

Analysis of 16S rDNA sequences of 24 strains resulted in further division into two groups. The first group included *B. solanacearum* biovar 3, 4, and 5 strains and the second group included biovars 1 and 2. Distance-based and parsimony-based trees were the basis for further separation into subdivisions 2a and 2b. An aberrant strain ACH0732 isolated from tomato in the Northern Territory of Australia had a 16S rDNA sequence and protein profile-like strains in biovars 3 and 4 but was phenotypically like biovar 2 strains. The positioning of this aberrant strain based on dendrograms created from different methodologies is summarized in Table 2.

## 8. 1995: Transfer of *Burkholderia solanacearum* into the New Genus *Ralstonia*

*Burkholderia solanacearum* and *B. pickettii* were like each other but differed from the five other species in the genus *Burkholderia* [51]. Moreover, the dendrogram of auxanographic tests showed a close relationship between *B. solanacearum, B. pickettii*, and *Alcaligenes eutrophus* [55]. Two kinds of ornithine-Lipids, OL-1 and OL-2, were characteristic of cellular lipids of *Burkholderia* species but these were lacking in *B. picketii, B. solanacearum,* and *B. eutropha* strains, and failure to utilize galactose, mannitol, mannose and sorbitol further distinguished them from *Burkholderia* species. Based on key differences between these strains and typical *Burkholderia* strains, the description of the genus *Burkholderia* was revised and the new taxa, *Ralstonia* gen. nov., *Ralstonia pickettii* comb. nov, *Ralstonia solanacearum* comb. nov., and *Ralstonia eutropha* comb. nov, were proposed [56]. The genus *Ralstonia* was named in honor of the bacteriologist Ericka Ralston who, along with Palleroni and Doudoroff, was the first to describe *Pseudomonas picketti* [57]. Ralston et al. had also shown that the relationship between the *P. pickettii* and *Pseudomonas solanacearum* was based on DNA homology [57]. At this point, the heterogeneity of *R. solanacearum* was not yet fully explained and continued to be an area for future research [56].

## 9. 1994–1996: Diversity Studies of the *Pseudomonas/Burkholderia/Ralstonia solanacearum* Species Complex

Molecular studies confirmed wide diversity among *P. solanacearum* strains, leading to the term “species complex”, first proposed in 1994 for *Pseudomonas solanacearum* [58]. The term was modified by Taghavi et al. in 1996 to include *Pseudomonas syzygii* and BDB strains [54]. The latter strains appeared to be closely related to the species complex by 16S rRNA gene sequence analyses despite their phenotypic differences from *P. solanacearum*, which at that point was already named *Burkholderia solanacearum* [54]. *Pseudomonas syzygii* had similar nucleotide sequences with members of the complex but previously had been considered a separate entity because of its distinct phenotype [48,54].

Sequences of the intergenic spacer region for 19 strains of *P. solanacearum*, one BDB strain and one strain of *P. syzygii* corroborated the division of the species complex as proposed by Taghavi 1996 [54,58]. Three distinct groups were formed based on polygalacturonase and endoglucanase gene sequences. Further studies confirmed and expanded the diversity of the bacterial wilt pathogen, now included in the *Ralstonia solanacearum* species complex [59].

## 10. 2000: Identification of the African Sub-Division

Phylogenetic trees generated from PCR restriction fragment length polymorphism (RFLP), amplified fragment length polymorphism (AFLP), and sequencing of 16S rRNA and the *hrp* gene region had separated all *P. solanacearum* species into two major groups (Asian and American), corroborating previous analyses [36]. With the inclusion of many African strains (some biovar 1 and others biovar 2), three different approaches were used to resolve the ambiguous position of *R. solanacearum* strains originating from Angola, Madagascar, Reunion Island, or Zimbabwe [59]. PCR-RFLP analysis of the *hrp* gene region placed the African biovar 1 strains into the “Asiaticum” division, showing further differentiation between the African and American biovar 1 strains. The AFLP and 16s rRNA data placed the biovar 2 strains from Angola, Madagascar, Reunion Island and Zimbabwe closer to the “Americanum” strains. Thus, a new subdivision (2c) was described to accommodate these strains [60]. Partial sequencing of *hrpB* genes of 30 strains in the *Ralstonia* species complex concurred with the previous findings [59]. Different restriction patterns were obtained for *P. syzygii* and BDB, confirming the differences between these strains and other *R. solanacearum* strains [60].

## 11. 2002: First Whole Genome Sequence of the Reference Strain GMI1000: General Structure of the Chromosome and Megaplasmid

Publication of the first whole-genome sequence of *R. solanacearum* strain GMI1000 opened the way for further studies on population diversity, phylogenetics and comparative genomics of *R*. *solanacearum* in addition to research on secretion systems, pathogenicity and virulence factors [61].

This landmark study describing the structure, pathogenicity, and evolution of this strain confirmed that the genome was divided into two replicons. The large 3.7 Mb replicon in *R. solanacearum*, referred to as the “chromosome” was associated with basic cellular survival mechanisms including DNA replication, DNA repair, transcription, translation, and cell cycle functions [61]. The chromosome shares common genes with other species whereas the 2.1 Mb megaplasmid harbors a relatively large number of genes with obscure functions [61]. The presence of a large plasmid in *R. solanacearum* had been previously reported in 1982 by Rosenberg et al. using screening protocols developed by Eckhardt [62,63]. The plasmid was detected by visualization of a slowly migrating band with a molecular weight greater than 450 × 10^6^ daltons observed in eight of the nine strains investigated. The megaplasmid was later shown to be the reservoir of genes that function in motility, host colonization, exopolysaccharide synthesis, and environmental adaptation [61]. Similar nucleotide composition in the protein-coding regions of both replicons was evidence that the chromosome and megaplasmid may have evolved together over a long period; thus, the plasmid, in a redundant state, may have retained several housekeeping gene functions essential for survival [64,65]. Both replicons harbor genes that function under diverse environmental conditions and may play a role in the survival of the bacterium [64].

Non-coding sequences that showed some homology with known proteins, termed Alternative Codon Usage Regions (ACURs), were present in strain GMI1000 [61]. Such regions were observed in 93 different sequence locations throughout the *R. solanacearum* genome and were characterized by large differences in base composition [66] (the average base composition for the entire genome was 67% G + C with variations ranging from 50 to 70% G + C content). Nearly half of the ACURs contained encoded insertion sequences, mobile elements, open reading frames, and genes encoding effector proteins [66]. The inclusion of encoded sequences in the ACUR region suggested that horizontal gene transfer had occurred in the species. Furthermore, the presence of truncated insertion sequences and possible pathogenicity islands (PAIs) may be indicators of rapid evolution in the genome [67]. The conjugative transposon site, recombinational hotspots, and nearly perfect tandem duplication present a strong case for horizontal gene transfer and possibly important evolutionary changes in the genomic composition [61].

Fifteen of 40 candidate genes responsible for pathogenicity had a G + C content that differed from the average G + C content of strain GMI1000 (67%), suggesting that these genes were acquired following horizontal gene transfer [61]. The 30 kb regions flanking the *hrp* gene cluster were devoid of insertion sequences and showed similarities in the G + C content with an ACUR. Researchers suggested that the flanking regions with virulence-related genes may have co-evolved with the core genome of *R. solanacearum* [61]. Sets of genes encoding hemagglutinin-related genes and a subclass of Type 3 Secretion System (T3SS)-dependent effectors were variable among strains. Formerly known as avirulence factors, these genes may contribute to host specificity traits [65,68]. The presence of ancestral T3SS, variable virulence genes, and horizontally acquired elements make *R. solanacearum* a highly successful pathogen in diverse environments, causing disease on a wide range of hosts.

Genetic transfer between bacteria may favor adaptation to different environmental conditions as well as modify the host range [69,70]. Recombination and horizontal gene transfer no doubt play important roles in determining the evolution of pathogenic bacteria and the tendency of bacteria to exchange genetic materials between distantly related species can make species definition ambiguous.

Similarities in the presence of pathogenicity and essential genes, nucleotide percentage and distribution patterns, and use of codons indicated that the chromosome and megaplasmid in *R*. *solanacearum* have coevolved over time [61,64,65]. Bacterial functions associated with motility, virulence, and resistance are associated with both replicons [61,71]

## 12. 2005: Introduction of a Phylotype-Based Classification of the *Ralstonia solanacearum* Species Complex

Successive molecular advances made through analysis of t-RNA consensus primers, 16 s rRNA sequences, PCR-RFLP, and AFLP profiles divided the RSSC into major phylogenetic groups [57,59,60]. Sequence analysis of the ITS region later formed the basis for the separation of the RSSC into four distinct groups, and a phylotype-based classification system was proposed [72,73]. The four phylotypes were further subdivided into sequevars, which consist of groups of strains showing high similarity based on partial sequences of the endoglucanase (*egl*) gene [72,73,74]. Different clonal lines within the sequevars were further differentiated using genomic fingerprinting methods such as rep-PCR, random amplification of polymorphic DNA (RAPD), AFLP, or pulsed-field gel electrophoresis (PFGE). The phylotypes, along with their respective phenotypic characteristics, are presented in Table 3.

**Figure 2:**
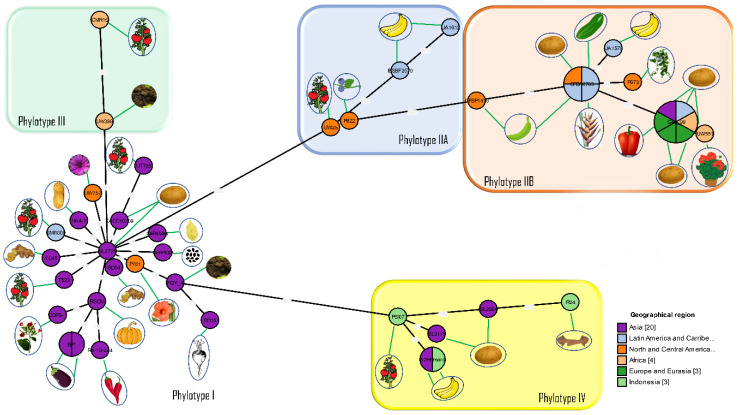
Phylogenetic relationships in the *Ralstonia solanacearum* species complex. Minimum spanning tree V2 was generated from concatenated sequences of six chromosomal housekeeping genes (*adk, dnaA, gap, gdh, gyrB* and *rplB*) and two megaplasmid genes (*hrpB* and *egl*) using GrapeTree software [75]. The number of compartments within a circle indicates the number of strains in the group. The number between any two lines is the distance between two strains calculated using the same software. The same strains were used in the ClonalFrame analyses. The details of the strains are provided in Supplementary Table S1. Note that reference strain GMI1000 originally isolated from French Guyana is in Phylotype I and clusters with strains of Asian origin.

The introduction of phylotype and sequevar as new standards for classification led to further comparisons of phylogenetic relationships among the worldwide population of *R. solanacearum* strains [72,74]. Most strains had previously been characterized by race and biovar and now had to be re-evaluated before conclusions could be made regarding their phylogenetic relationships. The relationships between phylotypes and their hosts of origin are shown in Figure 2.

## 13. 2006–2007: Core Genes and, Pathogenicity Determinants

An analysis of genetic diversity of 17 *R. solanacearum strains* using GMI1000 as the reference strain showed that only 53% of 5,074 genes from GMI1000 were in the core genome whereas 46% represented an approximate set of variable genes; the majority of the genes responsible for pathogenicity were core genes [65]. Variable genes were organized into genomic islands of two types, one of which had no counterpart in the core genome and may have been acquired from foreign genomes through lateral gene transfer. The second type had a GC content closer to the core genome. Ancestral genes may be lost by mutation during evolution or acquired through lateral gene transfer and subsequently transferred between strains in different phylotypes through vertical gene transfer [65]. In contrast to the vertical transfer of genetic elements in ACUR, prophage/insertion sequences appeared to have been transferred horizontally between the populations [65].

The stable and conserved nature of the housekeeping genes associated with the basic cellular functions also could be used to track the evolutionary forces acting on the interaction between bacteria and their hosts [76]. Housekeeping genes in the core genome are usually indispensable for bacterial survival and evolve slowly [76]. In contrast, the variable “flexible” or “accessory” genomes are dispensable, although they may affect the fitness and adaptation of bacteria [71].

Exopolysaccharides have an important role in the virulence of *R. solanacearum* [77,78]. The extracellular polysaccharide (Eps) operon is responsible for coding the proteins necessary for the biosynthetic pathway of EPS I [78,79]. The involvement of 10 regulatory promoters and more than 3 activation signals demonstrated the role of EPS I for inducing wilt by *R. solanacearum* [79,80]. The massive production of EPS I is controlled by the complex intricate operon in the EPS I pathway and is associated with distinguishing features between strains [79]. Additional pathogenicity determinants and their functions are described in Table 4.

## 14. 2010: Whole-Genome Analyses Further Highlight the Genomic Diversity across the Different Phylotypes

**Figure 3:**
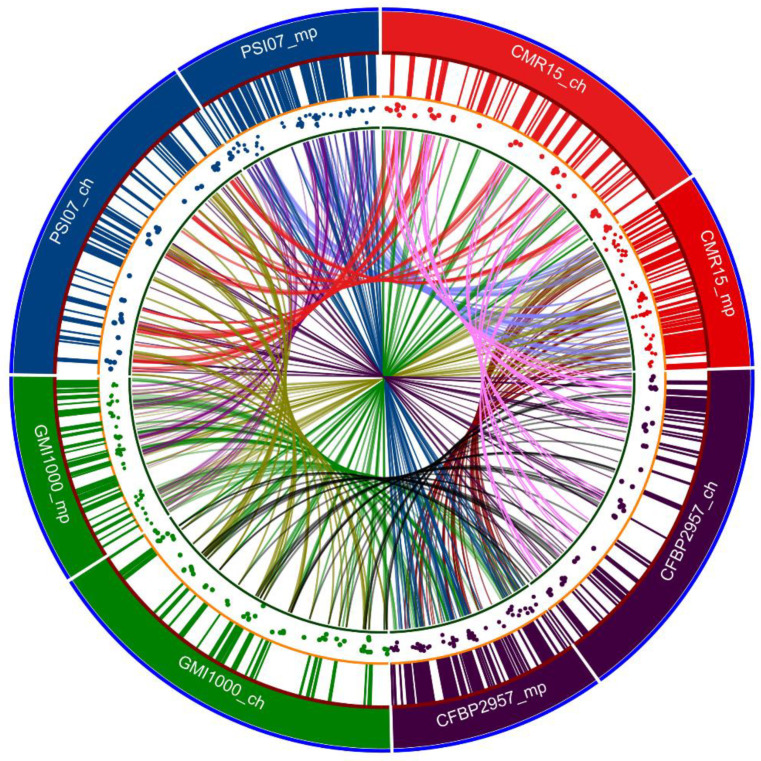
Circa plot showing the presence of predicted genomic islands in representative strains for each Phylotype of the RSSC. Track 1 (outer circle) shows the relative size of respective genomes: Phylotype I (GMI1000), Phylotype II (CFBP2957), Phylotype III (CMR15) and Phylotype IV (PSI07). The Ch tag stands for the chromosome and the mp tag stands for the megaplasmid. Approximate size of the replicons are: CMR15: ch—3.59 Mb, mp—2 Mb; CFBP2957: ch—3.42 Mb, mp—2.2 Mb; PSI07: ch—3.52 Mb, mp—2.09 Mb; GMI1000: ch—3.71 Mb, mp—2.1 Mb. Track 2 shows the relative positions of predicted genomic islands in their respective genomes. Track 3 is a scatter plot showing the GC content of the virulence gene coding sequences in the genome. Track 4 is a ribbon plot showing the links between the genomic islands in each genome. The image was generated using supplemental data of Remenant, et al. [86]; Island Viewer 4 software was used to predict genomic islands present in the GMI1000 genome retrieved from NCBI GenBank genome database. The Circa plot was created with Circa software (http://omgenomics.com/circa).

Remenant et al., 2010, 2011 conducted whole-genome comparative analysis of strains from each phylotype, including GMI1000, CFBP2957, IPO1609, and Molk2 CMR15, PSI07 [86,87]. Data from three completely sequenced tomato strains, CFBP2957, CMR15, PSI07 isolated in French West Indies, Cameroon, and Indonesia, respectively, were compared to data from pre-existing completely sequenced strains GMI1000, IPO1609, Molk2. They concluded that phylotype IV strains were sufficiently different to warrant classification as a separate species, which they named *R. haywardii* and they proposed that only phylotype II strains be retained as *R. solanacearum*, whereas phylotype I and III strains were proposed as a new species, *R. sequeirae.* Predicted genomic islands in representative strains for each phylotype of the RSSC are shown in Figure 3. The characteristics and origins of the reference strains from each phylotype are shown in Table 5.

The core genomes, dispensable genomes, and strain-specific genomes were 28%, 39% and 33% of the pan-genome, respectively [86]. Strain-specific genes encoding proteins with uncertain function ranged from 73 to 84% [86]. The density of the genomic islands was twofold higher on the megaplasmid of strains CFBP2957, CMR15, and PSI07 than on the chromosome [86]. A toxic operon *rhi* found on the megaplasmid of strains CFBP2957 and PSI07 was thought to have been acquired from *Burkholderia rhizoxina* and *Pseudomonas fluorescens* through natural transformation [95,96,97]. An additional *R. solanacearum* strain Po82 isolated from potato in Mexico also contained nearly all the *rhi* genes [98]. This strain was also pathogenic to banana and solanaceous crops. Phylogenetic analysis of different virulence factors of these six strains showed frequent addition and deletion events in the genomes of these strains. The presence of a plasmid was also reported in strains CMR15 and PSI07 [86]. The type III effector HopAF1 was reported in both *R. solanacearum* strain Po82 and *Xanthomonas* banana wilt strain *X. campestris* pathovar *musacearum* 4381 [98,99]. Likewise, the organization of Type IV effector genes on strain CMR15 of *Ralstonia* and virB cluster of pXAC64 plasmid in *Xanthomonas citri* pv. *citri* (strain 306) was nearly identical [86,100].

Genome shrinkage with larger deletions and subsequent gene loss may be the consequence of selective pressure to colonize different hosts [101,102]. For example, BDB and *Ralstonia syzygii* strains specific to banana and clove, respectively, are disseminated by insect vectors (xylem feeding *Hindola* spittlebugs), which reduce their competitive behavior [87]. The megaplasmids in BDB strain 229 and *R. syzygii* strain R24 are considerably smaller than the megaplasmids in other *R. solanacearum* strains [87]. Genome shrinkage in both these species may be attributed to the limited host species of these strains [87]. Strains R229 and R24 were devoid of a plasmid pRSI13, unlike the Phylotype IV strain PSI07, whereas the rhizoxin (*rhi*) operon was present on both the BDB and Phylotype II strain CBP2957 [86,87]. As the Phylotype II (CBP2957) and Phylotype IV (BDB) strains form a distinct division, Remenant et al. suggested that extrachromosomal gene transfer of *rhi* operon into the common ancestor of these two Phylotypes II and IV had a role in the evolutionary makeup [87]. The absence of the *fliC and fliT* genes in the genome of these strains explains the lack of motility in the strains R229 and R24 [50,87]. The average nucleotide identity (ANI) between completely sequenced Phylotype IV strains R229, R24 and PSI07 was above 98% [87]. As the DNA–DNA hybridization level of 70% is equivalent to 95% ANI value, the three species BDB, *R. syzygii* and the other Phylotype IV strains were proposed to be single genomic species [87].

## 15. 2012: Evolutionary History and Contrasting Recombination Patterns among Phylotypes

Multilocus sequence analysis was also used to unravel complex evolutionary patterns within the RSSC. In an evolutionary study of 58 *R. solanacearum* strains belonging to four phylotypes, five house-keeping genes and three virulence-related genes were analyzed by multilocus sequence typing (MLST). A high level of polymorphism was observed among alleles present in both the chromosome and the megaplasmid [76]. The housekeeping genes were variable for phylotype III strains, whereas these genes were highly clonal in the other phylotypes [76]. Furthermore, the *egl* genes involved in the degradation of cell wall products and the *hrpB* gene involved in the Type III secretion system showed high levels of recombination in phylotype III and phylotype IV strains.

**Figure 4:**
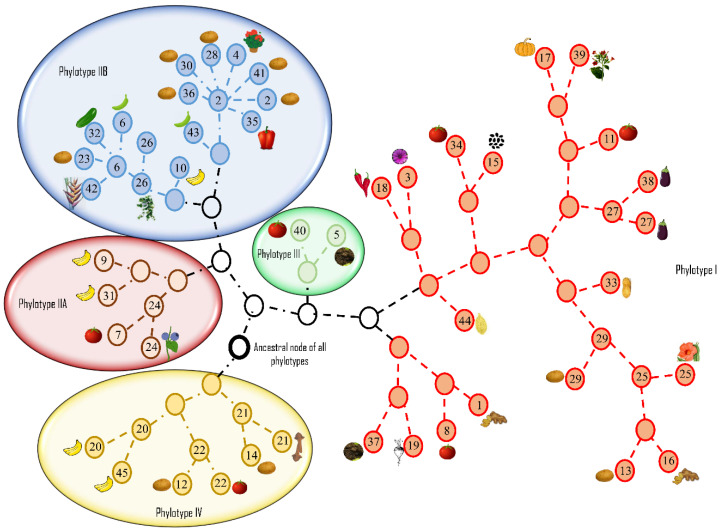
Graphviz network representing the genealogy of *Ralstonia solanacearum* species complex (RSSC) strains, representing geographically distinct phylotypes, generated using ClonalFrame v 1.0 [104]. Eight genes (*adk, dnaA, gap, gdh, gyrB, rplB, hrpB* and *egl*) were concatenated and aligned using progressive MAUVE alignment plugin [105]. The aligned sequences were used as input for ClonalFrame. The strain numbers inside each circle correspond to strains listed in Supplemental Table S1. Circles without numbers represent an unknown parental node for strains in that phylotype. The circle with the heavy black border is the theoretical ancestral node for all phylotypes. Plant host images adjacent to each circle represent the hosts from which the strains were isolated. Gene sequences for each strain were extracted from the NCBI GenBank database.

The high level of diversity in *R. solanacearum* was highlighted by the differences between the nucleotide composition, host range, and adaptability. Castillo and Greenberg provided two possible explanations for diversity within otherwise clonal populations: First, the populations may have evolved separately in geographical isolation, resulting in a distinct population structure for each phylotype [71,76]. The recombination analyses showed that the major and minor parents of a recombinant sequence in phylotypes III and IV were from their own respective subpopulations from the same geographical niche and not from phylotypes I (Asia) or II (America) [76]. A second explanation hinged upon the presence of rare genotypes that recombine at high rates and some emerge as clones through selective advantage and compete with the preexisting subpopulations to acquire a geographical niche [76,103]. Strains in phylotypes III and IV were the most diverse, suggesting that rare genotypes may exist, whereas the phylotypes I and II have clonal complexes that may have developed after acquiring genes that confer selective advantages over the original genotypes [76]. As might be expected, geographical isolation and spatial distance appeared to be the driving force in shaping the population structure of this species. The network representing the genealogy of RSSC strains is shown in Figure 4.

In a 2012 study involving 89 strains representing a broad geographic distribution, Wicker separated RSSC into eight clades with four lineages (phylotypes) and eight clades based on distinct evolutionary patterns [8]. Phylotype IIA contained clades 2 and 3 while phylotype IIB contained clades 4 and 5. Clades 1 and 6 were included in phylotypes I and III, respectively. Finally, phylotype IV consisted of clades 7 and 8 [8]. Studies of demographic histories *R. solanacearum* strains and recombination patterns involving seven housekeeping genes and two virulence-associated genes revealed that recombination occurred in seven out of nine genes [8]. Phylotype IV appeared to be the main donor for inter-phylotype gene exchange [8]. Phylotypes III and IV were found to be recombinogenic, highlighted by the predominant effect of recombination in shaping the evolutionary history of these groups (r/m value greater than 1). Likewise, the relative rate of recombination compared to mutation was higher in phylotypes I, III, IV (ρ/θ value greater than 1). The phylotype IIA strain group that originated in Northern Latin America and the Caribbean (described as clade 3 by Wicker et al.) was diverse and recombinogenic. The phylotype IIA subgroup showed recombinogenic population structure in contrast to the clonal IIB group, as shown by r/m statistics. [8].

The extent of recombination was not easily determined by comparing results of Castillo and Greenberg [76] with results of Wicker et al. [8], as the strains included in each study differed; nevertheless, conclusions regarding mutation and linkage patterns were consistent between the two studies [8,76]. Wicker et al. [8] suggested that the Australian/Indonesian region was the most probable origin for *R. solanacearum* due to the diversity, gene flow, topology, and branch length characteristics, which were in concordance with previous findings by Fegan [8,73]. Phylotype I strains harbor specific genetic elements that may have an important role in their worldwide dissemination and infection of new hosts such as woody perennials. Similarly, Phylotype IIA strains showed worldwide distribution [8].

## 16. Pathogenicity Functions Elucidated through Genomic Studies

Genomic studies of function, regulation, and pathogenicity provided information that eventually led to further reclassification of *R. solanacearum* strains. This pathogen has been used as one of the model systems for studies related to soil survival in the saprophytic phase, adaptation to new hosts and pathogenicity—including the production of pectinolytic enzymes, cell wall degradation, production and regulation of virulence factors. Exopolysaccharides were known to have an important role in virulence of *R. solanacearum* since very early studies of etiology by E.F. Smith [22] but genetic information has helped to unravel a very complex host–pathogen relationship. Genes related to key bacterial functions such as survival, saprophytic competition, adaption, chemotaxis, and infection have been thoroughly reviewed by Denny and Huang (1993); Shell, (2000); Genin, 2002, 2004; Jacobs and Allen, (2016) [4,66,67,79,106,107,108,109,110].

## 17. 2013: Gene Gain and Loss Contributing to Adaption and Bacterial Fitness

Cluster analysis of orthologous genes showed that the clusters with translation, ribosomal structure, and biogenesis activity were the most stable in *R. solanacearum*, whereas the genes involved in motility, transcription, lipid transport, and metabolism showed high mobilities [91]. Gene clusters associated with pathogenicity and adaptation were highly unstable. The gene gain or loss varied depending on the location of the genes on the chromosome.

Genes in the megaplasmid showed high gain or high loss compared to those in the chromosome [91]. However, chromosomal genes present in the “cell motility” class showed high gain in the chromosome compared to the megaplasmid. Thus, the differential gene gain and loss in both replicons suggest a variable contribution for adaptation and bacterial fitness [91]. Hot spots are genomic regions having higher single nucleotide variations (SNVs) whereas the cold spots have very few variations [106]. The hot spots were dominant in the megaplasmid, indicating higher gene variation, whereas cold spots were usually present on the chromosome [91]. No autocorrelation was found in the distance between the hotspots and the Insertion Sequence (IS) elements, suggesting that IS played no role in the clustering of hotspots and cold spots [91]. Horizontal gene transfers occurred frequently between the Molk2 banana Phylotype IIB strain and the two tomato strains, GMI1000 and CFBP2957 in Phylotypes I and IIA, respectively [91].

A unified nomenclature for the Type III effector (T3E) proteins was proposed by Peeters et al. [111] to bring consistency and avoid the use of dual names for single effectors. They proposed renaming 94 orthologous T3E genes, giving each a uniform generic name. The effectors in *R. solanacearum* were designated as Rip (*Ralstonia* Injected Proteins) to simplify the nomenclature and 2 groups of conserved effectors were later identified in 10 strains [111,112]. In addition to the generic name (*Rip*) for the genes, some portion of the previous name was incorporated into the new name for ease of identification (AvrA was changed to RipAA; PopP1 to RipP1). The GALA gene family was designated by a G extension, as in RipG1, RipG2 to RipG8 [111].

In some plant pathogenic bacteria like *Xanthomonas axonopodis*, the strains with similar T3E repertoires were associated with the same pathovar/host despite being phylogenetically distantly related [113]. Furthermore, there are numerous studies suggesting the primary role of T3Es in host specificity of *Pseudomonas syringae* at both pathovar–species and race–cultivar levels [114,115,116,117]. Thus, the comparative assessment of key pathogenicity related factors like T3Es can be helpful in the designation and differentiation of pathovars. However, it is important to note that the correlation between the T3E and host specificity does not hold true in all the plant pathogenic bacteria. In a study by Pensec et al. [118], the distribution of T3SS effectors was not significantly different among the phylotypes in RSSC. Strains belonging to the same phylotype and Type III repertoire group showed different virulence phenotypes and vice-versa.

The discovery of cold-tolerance genes or cool temperature virulence factors in R3bv2 strains was also revealed by comparative transcriptome analysis [85]. The adaptation of the R3bv2 strains to cooler temperature conditions was due to upregulation of a mannose–fucose binding lectin LecM, a specific quorum sensing-dependent protein, AidA, and a hypothetical protein AidC [85].

## 18. 2014–2020: Division into Three Genomic Species, Phylogenomics and Effector Repertoires

**Figure 5:**
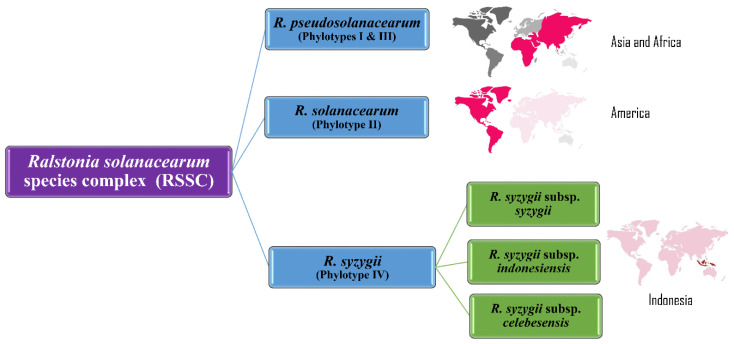
Taxonomic revision of *Ralstonia solanacearum* species complex phylotypes into three species: *R. pseudosolanacearum* (origin: Asia and Africa), *R. solanacearum* (origin: America) and *R. syzygii* (origin: Indonesia).

In a multifaceted polyphasic analysis of 68 strains from four phylotypes, Safni et al. [119] proposed further taxonomic revision of the diverse group of strains in phylotype IV. Phenotypic characterization including whole-cell fatty acid composition analysis, DNA base composition, DNA–DNA hybridization, ITS and *egl* gene analysis led to a proposal to further amend the species complex (Figure 5).

A complementary phenotypic analysis of strains representing all four phylotypes revealed that phylotype I and III strains utilized nitrate as an energy source under anaerobic conditions, unlike the phylotype II and IV strains [3]. Proteomic analysis of 73 bacterial strains using mass spectrometry also supported differentiation into three distinct species [3].

Prior et al. used the denitrification assays based on Dalsing et al. [120] whereas Safni et al. [119] used the method of Hayward in 1964 for denitrification assays [3,31,119]. The use of different assays for denitrification may explain the differences in utilization of nitrate energy sources by different phylotypes [3,119]. The Maximum Unique Matches index (MUMi) and Average Nucleotide Identity (ANI) analyses were used to delineate the species of the RSSC and supported the division of the species complex into three distinct groups, concurring with results of DNA–DNA hybridization studies [3,119]. Results from these analyses confirmed that the phylotype II strains clustered into a single species, *R. solanacearum*; phylotypes I and III comprised a second species, *R. pseudosolanacearum*, with two subgroups (IIA and IIB); the phylotype IV strains were classified as a separate species, *R. syzygii*, with three subspecies, *syzygii*, *indonesiensis*, and *celebesensis* [3,119].

Comparative genomic and transcriptomics analysis shows the adaptation of closely related populations of the RSSC to distinct host ranges. This was particularly relevant in the study of potato brown rot strains, moko strains, and strains non-pathogenic to banana (NPB). Each of these groups has closely related populations that have thrived well in hosts of distinct geographical locations [121]. Genes responsible for host range adaptations were minimal in clades containing brown rot strains, moko strains, and NPB. The moko strain UW163 (phylotype IIB) and the NPB strain IBSBF 1503 were closely related based on genomic content, but the latter strain was unable to infect banana and apparently had gained the ability to infect members of the Curcurbitaceae. These host range differences were associated with differences in their transcriptomic profiles, which differed in pathogenicity studies. Gene expression was convergent under similar environmental conditions, whereas virulence gene expression depended on the inoculated host [121]. The *ripAA* genes lost in the NPB during the divergent evolution from the Moko IIB lineage conferred resistance to the tomato strain, GMI1000 [122]. This suggests the *ripAA* may play a role as an effector for inducing banana wilt by moko strains. A strain, however, can have an avirulence factor conferring resistance in the host range of NPB strains excluding the host range of moko strains [122]. Comparative genomic analyses of moko strains and brown rot strains revealed 134 conserved hypothetical genes in brown rot strains that shared homology with soil-inhabitors and plant pathogenic bacteria. These genes may be involved in bacterial adaptation to unique hosts and different environmental conditions [122].

The use of multilocus sequence analysis (MLSA), proteomic analysis, DNA–DNA hybridization (DDH), next generation sequencing techniques have elucidated evolutionary patterns and pathogenicity-related gene regulatory mechanisms in this diverse and heterogeneous pathogen. However, positioning and classification of unique strains in the RSSC, such as ACH732, remains uncertain [8,53,54,72]. The *R. syzygii* strain DTP602 also is considered a misnomer based on ANI and DDH values and re-evaluation of its classification has been proposed [123].

In recent years, whole-genome sequencing-based phylogenomic studies—providing a comprehensive understanding of genome biology and constituents—are increasing [124,125,126,127]. Whole genome-based comparative genomic analysis is an advanced approach for locating the function of specific unknown genes, tracing evolutionary patterns based on genome organization, homologous recombination events, and taxonomic positioning of strains. Models for host specificity studies are based on effector repertoires, functional diversification, and effector-triggered immunity. These studies are significant, especially with respect to effector proteins-triggered elicitation and suppression of plant immunity [128,129]. In a recent study, Nakano and Mukaihara [130] revealed that *R. solanacearum* affects plant pattern-triggered immunity (PTI) using multiple effector proteins and modulates jasmonate signaling to stimulate infection. Specific genes within a whole-genome reference strain can be further analyzed to determine pathogen signaling in the environment, virulence pathways and pathogen adaptation to host defense mechanisms. The increasing genome database has made a significant contribution to predicting the role of genes in host adaptation and virulence and their evolutionary history [125]. As of a current NCBI Genome Assembly and Annotation report (September 2020), 93 strains of *R. solanacearum* have been completely sequenced, 15 additional strains were in chromosome level, 64 strains were in scaffold state and 45 were in contig phase (Table 6). The *R. syzygii* subsp. *indonesiensis* genomes have been included under the *R. solanacearum* genome in the NCBI GenBank database. Two genomes were found for *R. syzygii* subsp. *celebesensis*: A2-HR Mardi (CP019911.1) and R229 (FR854067.1). One genome was found for *R. syzygii* subsp. *syzygii*: R24 (FR854088.1).

The host range of one bacterial population may overlap with another and, in this regard, comparisons of genetic similarities and differences aid in understanding their evolutionary relationships. As virulence and host adaptational traits in bacteria are generally acquired through horizontal gene transfer, comparative genomic analyses provide insight into the ancestral and recent recombination events that may predict their future evolutionary trajectories. One study showed that the plasmid PRSC35 in a RSSC African strain CMR15 was broadly syntenic with many other plant pathogenic bacteria, like *X. citri* pv. *aurantifolli*, *Pseudomonas putida*, *X. euvesicatoria* [86]. Likewise, the Type IV secretion system genes in CMR 15 strain had nearly the same organization as PXAC64 of *X. citri* pv. citri [100]. This suggests the important role of the Type IV secretion system in the virulence mechanism of the pathogen. Thorough understanding of the pathogenomics and evolutionary history of a pathogen is crucial in developing effective and sustainable control strategies. Multiple studies have been conducted to explore the different control methods and resistance for the bacterial wilt pathogen and readers are directed to studies by Yuliar et al., Yang et al., Wei et al., Chen et al., Elsayed et al., Zheng et al., Nguyen and Ranamukhaarechchi, Fuijwara et al., Lemesa and Zeller, Mansfield, Huet, Deslandes et al., Wang et al., for further reading on resistance and control of the bacterial wilt disease [131,132,133,134,135,136,137,138,139,140,141,142,143,144,145]. 

## 19. Conclusions and Future Research

Significant research advances have been made over the past 124 years on the bacterial wilt pathogen, first described as a Gram-positive rod, *Bacillus solanacearum*. Frequent name changes have occurred as methodology transitioned from phenotypic, biochemical, and molecular studies, to genomics and functional genomics. The diversity, wide host range and geographical distribution of *R. solanacearum* resulted in its inclusion in a “species complex” following genomic analyses of elucidated phylogenetic relationships among strains. Lower costs for whole-genome sequencing have enabled researchers to go beyond MLST-based analyses to describe the diversity and evolutionary relationships among strains. In 2015, there were only 10 completely sequenced genomes. In 2020, 18 years after the publication of the first genome sequence for *R. solanacearum*, 217 whole genome sequences have been published in the NCBI GenBank database for the three currently described species in the RSSC, *R. solanacearum, R. pseudosolanacearum* and *R. syzygii*. Genome resources for RSSC strains with different levels of assembly (complete, chromosome, scaffold and contig) are available in the NCBI GenBank database (Table 6). While most of the genomic resources are available for phylotype I and IIB strains, fewer are available for phylotype IIA, III and IV strains. Interestingly, phylotype I strains were the least diverse while phylotype III and IV strains were the most diverse, as shown by Wicker et al. [8]. With the advent of less expensive and convenient whole genome sequencing technologies like Oxford Nanopore and Ion torrent (Supplemental Table S2), whole genome based comparative genomics studies on more phylotype III, IV and IIA strains will reveal interesting facts on genome composition, phylogenomics, evolutionary dynamics and opportunities for more host–microbe interactions, transcriptomics and host adaptation studies. The broad host range and diversified distribution of RSSC throughout the world will no doubt lead to the discovery of new strains in the future, leading to further taxonomic modifications of RSSC. Next generation sequencing has set a rapid pace while advancing the taxonomic and phylogenomic research. A comprehensive analysis of the genomic biology of this pathogen is still needed to understand the recombination events and evolution of this devastating pathogen. To resolve the evolutionary events (both ancestral and recent) of this pathogen, it is important to determine how RSSC genomic constituents, genes, or gene clusters, react in different niches associated with diverse hosts, leading to delineation of species.

## Figures and Tables

**Table 1:** Classification of *Pseudomonas solanacearum* strains into biovars based on oxidation of three disaccharides and three hexose alcohols [31,32].

Biotype ^a^	Disaccharides			Hexose Alcohols			References
	Lactose	Maltose	Cellobiose	Dulcitol	Mannitol	Sorbitol	
1	−	−	−	−	−	−	[31]
2	+	+	+	−	−	−	[31]
3	+	+	+	+	+	+	[31]
4	−	−	−	+	+	+	[31]
5	+	+	+	−	+	−	[32] ^b^

^a^ Biotype is synonymous with biovar. Hayward initially described only four biovars. ^b^ Strains from mulberry were tentatively classified as race 4, biotype 5 by He et al., 1983, and later reclassified as race 5, biotype 5 [32].

**Table 2:** Relationship of an aberrant strain, ACH0732, based on dendrograms derived from different *Burkholderia solanacearum* gene sequences [53,54].

Method Used to Create the Dendrogram	Position of Strain ACH0732 in the Dendrogram
16S-23S rRNA intergenic spacer region sequences	Outside of all three clusters
Polygalacturonase gene sequences	Outside of all three clusters
Endogluconase gene sequences	Subdivision 2b
16S rDNA-based PCR	Division 1
ITS multiplex PCR	Subdivision 2b

**Table 3:** Phylotypes of the *Ralstonia solanacearum* species complex [72,74].

Phylotype	General Geographical Origin	Phenotypic Characteristics
I	Asia	Biovars 3, 4 and 5
II	America	Biovars 1, 2 and 2T ^a^
III	Africa and surrounding islands	Biovars 1 and 2T
IV	Indonesia	Biovars 1, 2 and 2T; *P. syzygii* and BDB ^b^

^a^ Biovar 2T = 2N; ^b^ Blood disease bacteria.

**Table 4:** Operons and gene products of significance in pathogenesis and genomic studies of the *Ralstonia solanacearum* species complex.

Operons/Gene Products	Name	Function	References
Eps operon	Extracellular polysaccharide	Production of EPS I	[78,79]
Cel, PME, PG	Cellulase, pectin methylesterase, cellobiosidase and polygalacturonases	Plant cell wall degradation	[79,81,82]
PehA	Pectin methylesterase	Exposes pectin to degradation by removing its methoxy groups	[79,83]
PehB	Endo-polygalacturonic acid esterase	Pectin degradation	[79,84]
PehC	Exo-polygalacturonic acid esterase	Pectin degradation	[79,81]
PehX	Endo-polygalacturonic acid esterase (like PehB)	Aids conversion of complex polygalacturonic acid and pectin into simple monomeric, dimeric, or oligomeric units	[79]
LecM	Mannose–fucose binding lectin	Contributes to cool temperature virulence of R3bv2 strains	[85]
AidA, AidC	Quorum-sensing protein and a hypothetical protein	Contributes to cool temperature virulence of R3bv2 strains	[85]

**Table 5:** Reference strains of *Ralstonia solanacearum*, *R. sygyzii*, and BDB: their characteristics and origins.

Strain No.	Race, Biovar, Phylotype ^a^	Host	Origin	Reference
GMI1000	R 1, bv 3, phylotype I	Tomato	French Guyana	[88]
K60	R 1, bv 1, phylotype IIA	Tomato	North Carolina, USA	[17]
CFBP2957	phylotype IIA	Tomato	French West Indies	[86,89]
IPO 1609	R 3, bv 2, phylotype IIB	Potato	The Netherlands	[90]
Molk2 ^b^	R 2, bv 1, phylotype IIB	Banana	Philippines	[65,91]
ACH0732	R1, bv 2	Tomato	Australia	[53]
R240	R1, bv N2	Potato	Nepal	[92,93]
R780	R1, bv N2	Potato	Indonesia	[53]
CMR15	phylotype III	Tomato	Cameroon	[86,94]
PSI07	phylotype IV	Tomato	Indonesia	[74,86]
R229	BDB ^c^ phylotype IV	Banana	Indonesia	[87]
R24	*R. syzygii* phylotype IV	Clove	Indonesia	[87]

^a^*Ralstonia solanacearum* unless otherwise indicated; ^b^ Molk2, was originally isolated from banana in Mindanao, the Philippines by A. Raymundo, unpublished; ^c^ Blood Disease Bacterium.

**Table 6:** Genome sequences of the *Ralstonia solanacearum* species complex (RSSC) available in the NCBI GenBank genome database highlighting the numerical distribution among different phylotypes. The levels of assembly (complete, chromosome, scaffold and contig) are categorized for each phylotype.

Phylotype	Complete	Chromosome	Scaffold	Contig	Total
I	67	6	37	18	128
III	1	1		1	3
IIA	1	3	7	6	17
IIB	11	4	20	18	53
IV	13	1		2	16
Total	93	15	64	45	217

## Supplementary Materials

The following are available online at https://www.mdpi.com/2076-0817/9/11/886/s1, Table S1: Strain information used to create ClonalFrame analyses and Minimum Spanning tree in Figure 4. Table S2: Description of Ralstonia solanacearum species complex strains available in the NCBI GenBank database with associated host, country of isolation and sequencing technology used.

## References

[1] Kannan V., Bastas K., Antony R. (2015). Plant pathogenic bacteria: An overview. Sustainable Approaches to Controlling Plant Pathogenic Bacteria.

[2] Hayward A.C. (1991). Biology and Epidemiology of Bacterial Wilt Caused by *Pseudomonas solanacearum*. Annu. Rev. Phytopathol..

[3] Prior P., Ailloud F., Dalsing B.L., Remenant B., Sanchez B., Allen C. (2016). Genomic and proteomic evidence supporting the division of the plant pathogen *Ralstonia solanacearum* into three species. BMC Genom..

[4] Genin S. (2010). Molecular traits controlling host range and adaptation to plants in *Ralstonia solanacearum*. New Phytol..

[5] Van Overbeek L.S., Bergervoet J.H.W., Jacobs F.H.H., Van Elsas J.D. (2004). The low-temperature-induced viable-but-nonculturable State affects the virulence of *Ralstonia solanacearum* biovar 2. Phytopathology.

[6] Mansfield J., Genin S., Magori S., Citovsky V., Sriariyanum M., Ronald P., Dow M., Verdier V., Beer S.V., Machado M.A. (2012). Top 10 plant pathogenic bacteria in molecular plant pathology. Mol. Plant Pathol..

[7] Elphinstone J.G. (2005). The current bacterial wilt situation: A global overview. Bacterial wilt Disease and the Ralstonia solanacearum Species Complex.

[8] Wicker E., Lefeuvre P., De Cambiaire J.-C., Lemaire C., Poussier S., Prior P. (2011). Contrasting recombination patterns and demographic histories of the plant pathogen *Ralstonia solanacearum* inferred from MLSA. ISME J..

[9] Floyd J. (2007). New Pest Response Guidelines: *Ralstonia solanacearum* race 3 biovar 2. USDAAPHIS-PPQ, Emergency and Domestic Programs, Riverdale, MD. https://www.ippc.int/static/media/uploads/resources/new_pest_response_guidelines_ralstonia_solanacearum_race_3_biovar_2.pdf.

[10] National Potato Council (2020). 2020 Annual Potato Yearbook.

[11] AgMRC (2018). Tomatoes.

[12] Champoiseau P. (2009). Ralstonia solanacearum Race 3 biovar 2. From the Field to the Lab: Towards Accurate Identification of a Select Agent Pathogen.

[13] Smith E.F. (1896). A Bacterial Disease of the Tomato, Eggplant, and Irish Potato (Bacillus solanacearum n. sp.).

[14] Burrill T.J. Preliminary notes upon the rotting of potatoes. Proceedings of the 11th Annual Meeting Society for Promotion of Agricultural Sciences.

[15] Burrill T.J. Additional note on the rot of potatoes. Proceedings of the Washington Meeting of the Society for the Promotion of Agricultural Science.

[16] Halsted B.D. (1892). An Investigation of Tomato Blight, a Blight of Potatoes, Bacterial Melon Blight.

[17] Kelman A. (1953). The Bacterial Wilt Caused by Pseudomonas Solanacearum: A Literature Review and Bibliography.

[18] Smith E.F. The southern tomato blight. Proceedings of the American American Association for the Advancement of Science.

[19] Chester F.D. (1898). A preliminary arrangement of the species of the genus Bacterium. JAMA.

[20] Smith E.F. (1909). The Granville Tobacco Wilt.

[21] Rorer J.B. (1911). A bacterial disease of bananas and plantains. Phytopathology.

[22] Smith E.F. (1905). Bacteria in Relation to Plant Diseases.

[23] Bergey D.H. (1923). Manual of Determinative Bacteriology.

[24] Dowson W. (1943). On the generic names *Pseudomonas, Xanthomonas* and *Bacterium* for certain bacterial plant pathogens. Trans. Br. Mycol. Soc..

[25] Dowson W.J. (1949). Manual of Bacterial Plant Diseases.

[26] Breed R.S., Murray E.G.D., Hitchens A.P. (1948). Bergey’s Manual of Determinative Bacteriology.

[27] Savulescu T. (1948). Contribution a la classification des bacteriacees phytopathogenes. Rev. Appl. Mycol..

[28] Buddenhagen I., Sequeira L., Kelman A. (1962). Designation of races in *Pseudomonas solanacearum*. Phytopathology.

[29] Buddenhagen I., Kelman A. (1964). Biological and physiological aspects of bacterial wilt caused by *Pseudomonas solanacearum*. Annu. Rev. Phytopathol..

[30] Dye D.W., Bradbury J.F., Goto M., Hayward A.C., Leelliott R.A., Schroth M.N. (1980). International standards for naming pathovars of phytopathogenic bacteria and a list of pathovar names and pathotype strains. Rev. Plant Pathol..

[31] Hayward A.C. (1964). Characteristics of *Pseudomonas solanacearum*. J. Appl. Bacteriol..

[32] He L.Y. (1983). Characteristics of Strains of *Pseudomonas solanacearum* from China. Plant Dis..

[33] Aragaki M., Quinon V.L. (1965). Bacterial wilt of ornamental gingers (*Hedychium* spp.) caused by *Pseudomonas solanacearum*. Plant Dis. Rep..

[34] Pegg K., Moffett M. (1971). Host range of the ginger strain of *Pseudomonas solanacearum* in Queensland. Aust. J. Exp. Agric..

[35] Pan Z.C., Xu J.S., Prior P., Zhang H., Chen K.Y., Tian Q., Zhang L.Q., Liu L., He L.Y., Feng J. (2013). Development of a specific molecular tool for the detection of epidemiologically active mulberry causing-disease strains of *Ralstonia solanacearum* phylotype I (historically race 5-biovar 5) in China. Eur. J. Plant Pathol..

[36] Cook D. (1989). Genetic Diversity of *Pseudomonas solanacearum*: Detection of restriction fragment length polymorphisms with DNA probes that specify virulence and the hypersensitive response. Mol. Plant-Microbe Interact..

[37] Palleroni N.J., Doudoroff M. (1971). Phenotypic characterization and deoxyribonucleic acid homologies of *Pseudomonas solanacearum*. J. Bacteriol..

[38] Doi R.H., Igarashi R.T. (1965). Conservation of ribosomal and messenger ribonucleic acid cistrons in *Bacillus* species. J. Bacteriol..

[39] Dubnau D., Smith I., Morell P., Marmur J. (1965). Gene conservation in *Bacillus* species. I. Conserved genetic and nucleic acid base sequence homologies. Proc. Natl. Acad. Sci. USA.

[40] Palleroni N.J., Kunisawa R., Contopoulou R., Doudoroff M. (1973). Nucleic acid homologies in the genus *Pseudomonas*. Int. J. Syst. Bacteriol..

[41] Li X., Dorsch M., Del Dot T., Sly L., Stackebrandt E., Hayward A. (1993). Phylogenetic studies of the rRNA group II pseudomonads based on 16S rRNA gene sequences. J. Appl. Bacteriol..

[42] Byng G.S., Johnson J.L., Whitaker R.J., Gherna R.L., Jensen R.A. (1983). The evolutionary pattern of aromatic amino acid biosynthesis and the emerging phylogeny of pseudomonad bacteria. J. Mol. Evol..

[43] De Vos P., De Ley J. (1983). Intra- and intergeneric similarities of *Pseudomonas* and *Xanthomonas* ribosomal ribonucleic acid cistrons. Int. J. Syst. Bacteriol..

[44] De Vos P., Goor M., Gillis M., De Ley J. (1985). Ribosomal ribonucleic acid cistron similarities of phytopathogenic *Pseudomonas* species. Int. J. Syst. Bacteriol..

[45] De Vos P. (1980). Intrageneric and intergeneric similarities of ribosomal RNA cistrons of the genus *Pseudomonas* and the implications for taxonomy. Antonie van Leeuwenhoek.

[46] Seal S.E., Jackson L.A., Daniels M.J. (1992). Use of tRNA consensus primers to indicate subgroups of *Pseudomonas solanacearum* by polymerase chain reaction amplification. Appl. Environ. Microbiol..

[47] Welsh J., McClelland M. (1991). Genomic fingerprints produced by PCR with consensus tRNA gene primers. Nucleic Acids Res..

[48] Eden-Green S.J., Adhi E. (1987). Sumatra Disease of Cloves and Pseudomonas solanacearum.

[49] Eden-Green S., Sastraatmadja H. (1990). Blood Disease in Indonesia.

[50] Roberts S., Eden-Green S., Jones P., Ambler D. (1990). *Pseudomonas syzygii*, sp. nov., the cause of Sumatra disease of cloves. Syst. Appl. Microbiol..

[51] Seal S.E., Jackson L.A., Young J.P.W., Daniels M.J. (1993). Differentiation of *Pseudomonas solanacearum*, *Pseudomonas syzygii, Pseudomonas pickettii* and the Blood Disease Bacterium by partial 16S rRNA sequencing: Construction of oligonucleotide primers for sensitive detection by polymerase chain reaction. J. Gen. Microbiol..

[52] Yabuuchi E., Kosako Y., Oyaizu H., Yano I., Hotta H., Hashimoto Y., Ezaki T., Arakawa M. (1992). Proposal of Burkholderia gen. nov. and transfer of seven species of the genus *Pseudomonas* homology group II to the new genus, with the type species *Burkholderia cepacia* (Palleroni and Holmes 1981) comb. nov. Microbiol. Immunol..

[53] Fegan M., Taghavi M., Sly L.I., Hayward A.C., Prior P., Allen C., Elphinstone J. (1998). Phylogeny, diversity, and molecular diagnostics of *Ralstonia solanacearum*. Bacterial Wilt Disease: Molecular and Ecological Aspects.

[54] Taghavi M., Hayward C., Sly L.I., Fegan M. (1996). Analysis of the phylogenetic relationships of strains of *Burkholderia solanacearum*, *Pseudomonas syzygii*, and the Blood Disease Bacterium of banana based on 16S rRNA gene sequences. Int. J. Syst. Bacteriol..

[55] Gillis M., Van Van T., Bardin R., Goor M., Hebbar P., Willems A., Segers P., Kersters K., Heulin T., Fernandez M.P. (1995). Polyphasic taxonomy in the genus *Burkholderia* leading to an emended description of the genus and proposition of *Burkholderia vietnamiensis* sp. nov. for N2-Fixing isolates from rice in Vietnam. Int. J. Syst. Bacteriol..

[56] Yabuuchi E., Kosako Y., Yano I., Hotta H., Nishiuchi Y. (1995). Transfer of two *Burkholderia* and an *Alcaligenes* species to *Ralstonia* Gen. Nov. Microbiol. Immunol..

[57] Ralston E., Palleroni N.J., Doudoroff M. (1973). *Pseudomonas pickettii*, a new species of clinical origin related to *Pseudomonas solanacearum*. Int. J. Syst. Bacteriol..

[58] Gillings M., Fahy P. (1994). Genomic fingerprinting: Towards a unified view of the Pseudomonas solanacearum species complex. Bacterial Wilt: The Disease and Its Causative Agent, Pseudomonas solanacearum.

[59] Poussier S., Prior P., Luisetti J., Hayward C., Fegan M. (2000). Partial Sequencing of the *hrpB* and *Endoglucanase* genes confirms and expands the known diversity within the *Ralstonia solanacearum* species complex. Syst. Appl. Microbiol..

[60] Poussier S., Trigalet-Demery D., Vandewalle P., Goffinet B., Luisetti J., Trigalet A. (2000). Genetic diversity of *Ralstonia solanacearum* as assessed by PCR-RFLP of the *hrp* gene region, AFLP and 16S rRNA sequence analysis, and identification of an African subdivision. Microbiology.

[61] Salanoubat M., Genin S., Artiguenave F., Gouzy J., Mangenot S., Arlat M., Billault A., Brottier P., Camus J.C., Cattolico L. (2002). Genome sequence of the plant pathogen *Ralstonia solanacearum*. Nature.

[62] Eckhardt T. (1978). A rapid method for the identification of plasmid desoxyribonucleic acid in bacteria. Plasmid.

[63] Rosenberg C., Casse-Delbart F., Dusha I., David M., Boucher C. (1982). Megaplasmids in the plant-associated bacteria *Rhizobium meliloti* and *Pseudomonas solanacearum*. J. Bacteriol..

[64] Coenye T., Vandamme P. (2003). Simple sequence repeats and compositional bias in the bipartite *Ralstonia solanacearum* GMI1000 genome. BMC Genom..

[65] Guidot A., Prior P., Schoenfeld J., Carrère S., Genin S., Boucher C. (2006). Genomic Structure and Phylogeny of the Plant Pathogen *Ralstonia solanacearum* Inferred from Gene Distribution Analysis. J. Bacteriol..

[66] Genin S., Boucher C. (2004). Lessons learned from the genome analysis of *Ralstonia solanacearum*. Annu. Rev. Phytopathol..

[67] Genin S., Boucher C. (2002). *Ralstonia solanacearum*: Secrets of a major pathogen unveiled by analysis of its genome. Mol. Plant Pathol..

[68] Alfano J.R., Collmer A. (2004). Type III secretion system effector proteins: Double agents in bacterial disease and plant defense. Annu. Rev. Phytopathol..

[69] Cohan F.M. (2002). What are bacterial species?. Annu. Rev. Microbiol..

[70] Ochman H., Lawrence J.G., Groisman E.A. (2000). Lateral gene transfer and the nature of bacterial innovation. Nature.

[71] Sarkar S.F., Guttman D.S. (2004). Evolution of the core genome of *Pseudomonas syringae*, a highly clonal, endemic elant pathogen. Appl. Environ. Microbiol..

[72] Prior P., Fegan M. (2005). Recent developments in the phylogeny and classification of *Ralstonia solanacearum*. Acta Hortic..

[73] Fegan M., Prior P. (2005). How complex is the *Ralstonia solanacearum* species complex. Bacterial Wilt Disease and the Ralstonia solanacearum Species Complex.

[74] Fegan M. (2005). Bacterial wilt Diseases of Banana: Evolution and Ecology in Bacterial Wilt Disease and the Ralstonia solanacearum Species Complex.

[75] Zhou Z., Alikhan N.-F., Sergeant M.J., Luhmann N., Vaz C., Francisco A.P., Carriço J.A., Achtman M. (2018). GrapeTree: Visualization of core genomic relationships among 100,000 bacterial pathogens. Genome Res..

[76] Castillo J.A., Greenberg J.T. (2006). Evolutionary dynamics of *Ralstonia solanacearum*. Appl. Environ. Microbiol..

[77] Orgambide G., Montrozier H., Servin P., Roussel J., Trigalet-Demery D., Trigalet A. (1991). High heterogeneity of the exopolysaccharides of *Pseudomonas solanacearum* strain GMI 1000 and the complete structure of the major polysaccharide. J. Biol. Chem..

[78] Schell M., Denny T., Clough S., Huang J., Nester E.W., Verma D.P.S. (1993). Further characterization of genes encoding extracellular polysaccharide of *Pseudomonas solanacearum* and their regulation. Advances in Molecular Genetics of Plant-Microbe Interactions, Volume 2, Proceedings of the 6th International Symposium on Molecular Plant-Microbe Interactions, Seattle, WA, USA, July 1992.

[79] Schell M.A. (2000). Control of virulence and pathogenicity genes of *Ralstonia solanacearum* by an elaborate sensory network. Annu. Rev. Phytopathol..

[80] Huang J., A Schell M. (1995). Molecular characterization of the eps gene cluster of *Pseudomonas solanacearum* and its transcriptional regulation at a single promoter. Mol. Microbiol..

[81] González E.T., Allen C. (2003). Characterization of a *Ralstonia solanacearum* operon required for polygalacturonate degradation and uptake of galacturonic acid. Mol. Plant Microbe Interact..

[82] Kang Y. (1994). Dramatically reduced virulence of mutants of *Pseudomonas solanacearum* defective in export of extracellular proteins across the outer membrane. Mol. Plant Microbe Interact..

[83] Allen C. (1991). Cloning of genes affecting polygalacturonase production in *Pseudomonas solanacearum*. Mol. Plant Microbe Interact..

[84] Huang Q., Allen C. (1997). An exo-poly-alpha-d-galacturonosidase, PehB, is required for wild-type virulence of *Ralstonia solanacearum*. J. Bacteriol..

[85] Meng F., Babujee L., Jacobs J.M., Allen C. (2015). Comparative transcriptome analysis reveals cool virulence factors of *Ralstonia solanacearum* race 3 biovar 2. PLoS ONE.

[86] Remenant B., Coupat-Goutaland B., Guidot A., Cellier G., Wicker E., Allen C., Fegan M., Pruvost O., Elbaz M., Calteau A. (2010). Genomes of three tomato pathogens within the *Ralstonia solanacearum* species complex reveal significant evolutionary divergence. BMC Genom..

[87] Remenant B., De Cambiaire J.-C., Cellier G., Jacobs J.M., Mangenot S., Barbe V., Lajus A., Vallenet D., Medigue C., Fegan M. (2011). *Ralstonia syzygii*, the Blood Disease Bacterium and some asian *R. solanacearum* strains form a single genomic species despite divergent lifestyles. PLoS ONE.

[88] Boucher C.A., Barberis P.A., Demery D.A. (1985). Transposon mutagenesis of *Pseudomonas solanacearum*: Isolation of Tn5-induced avirulent mutants. Microbiology.

[89] Prior P., Steva H. (1990). Characteristics of strains of Pesudomonas solanacearum from the French West Indies. Plant Dis..

[90] Van Elsas J.D., Kastelein P., De Vries P.M., Van Overbeek L.S. (2001). Effects of ecological factors on the survival and physiology of *Ralstonia solanacearum* bv. 2 in irrigation water. Can. J. Microbiol..

[91] Lefeuvre P., Cellier G., Remenant B., Chiroleu F., Prior P. (2013). Constraints on genome dynamics revealed from gene distribution among the *Ralstonia solanacearum* species. PLoS ONE.

[92] Seal S., Taghavi M., Fegan N., Hayward A.C., Fegan M. (1999). Determination of *Ralstonia* (*Pseudomonas*) *solanacearum* rDNA subgroups by PCR tests. Plant Pathol. J..

[93] Smith J., Offord L., Holderness M., Saddler G. (1996). Genetic diversity of *Burkholderia solanacearum* (synonym *Pseudomonas solanacearum*) race 3 in Kenya. Appl. Environ. Microbiol..

[94] Toukam G.M.S., Cellier G., Wicker E., Guilbaud C., Kahane R., Allen C., Prior P. (2009). Broad diversity of *Ralstonia solanacearum* strains in Cameroon. Plant Dis..

[95] Coupat B., Chaumeille-Dole F., Fall S., Prior P., Simonet P., Nesme X., Bertolla F., Coupat-Goutaland B. (2008). Natural transformation in the *Ralstonia solanacearum* species complex: Number and size of DNA that can be transferred. FEMS Microbiol. Ecol..

[96] Loper J.E., Henkels M.D., Shaffer B.T., Valeriote F.A., Gross H. (2008). Isolation and identification of rhizoxin analogs from *Pseudomonas fluorescens* Pf-5 by using a genomic mining strategy. Appl. Environ. Microbiol..

[97] Partida-Martinez L.P., Hertweck C. (2007). A Gene cluster encoding rhizoxin biosynthesis in “*Burkholderia rhizoxina*”, the bacterial endosymbiont of the fungus *Rhizopus microsporus*. ChemBioChem.

[98] Xu J., Zheng H.-J., Liu L., Pan Z., Prior P., Tang B., Zhang H., Tian Q., Zhang L.-Q., Feng J. (2011). Complete genome sequence of the plant pathogen *Ralstonia solanacearum* strain Po82. J. Bacteriol..

[99] Studholme D.J., Kemen E., MacLean D., Schornack S., Aritua V., Thwaites R., Grant M., Smith J., Jones J.D. (2010). Genome-wide sequencing data reveals virulence factors implicated in banana xanthomonas wilt. FEMS Microbiol. Lett..

[100] El Yacoubi B., Brunings A.M., Yuan Q., Shankar S., Gabriel D.W. (2007). In planta horizontal transfer of a major pathogenicity effector gene. Appl. Environ. Microbiol..

[101] Mira A., Ochman H., Moran N.A. (2001). Deletional bias and the evolution of bacterial genomes. Trends Genet..

[102] Moran N.A. (2002). Microbial minimalism. Cell.

[103] Levin B.R. (1981). Periodic selection, infectious gene exchange and the genetic structure of *E. coli* populations. Genetics.

[104] Didelot X., Falush D. (2006). Inference of bacterial microevolution using multilocus sequence data. Genetics.

[105] Darling A., Mau B., Perna N. (2010). progressiveMauve: Multiple genome alignment with gene gain, loss and rearrangement. PLoS ONE.

[106] Brumbley S.M., Carney B.F., Denny T.P. (1993). Phenotype conversion in *Pseudomonas solanacearum* due to spontaneous inactivation of PhcA, a putative LysR transcriptional regulator. J. Bacteriol..

[107] Brumbley S.M., Denny T.P. (1990). Cloning of wild-type *Pseudomonas solanacearum phcA*, a gene that when mutated alters expression of multiple traits that contribute to virulence. J. Bacteriol..

[108] Denny T.P. (1990). Inactivation of multiple virulence genes reduces the ability of *Pseudomonas solanacearum* to cause wilt symptoms. Mol. Plant Microbe Interact..

[109] Genin S., Denny T. (2012). Pathogenomics of the *Ralstonia solanacearum* species complex. Annu. Rev. Phytopathol..

[110] Jacobs J.M., Allen C. (2016). Virulence mechanisms of plant-pathogenic Ralstonia spp.. Virulence Mechanisms of Plant-Pathogenic Bacteria.

[111] Peeters N., Carrère S., Anisimova M., Plener L., Cazalé A.-C., Genin S. (2013). Repertoire, unified nomenclature and evolution of the Type III effector gene set in the *Ralstonia solanacearum* species complex. BMC Genom..

[112] Deslandes L., Genin S. (2014). Opening the *Ralstonia solanacearum* type III effector tool box: Insights into host cell subversion mechanisms. Curr. Opin. Plant Biol..

[113] Hajri A., Brin C., Hunault G., Lardeux F., Lemaire C., Manceau C., Boureau T., Poussier S. (2009). A «Repertoire for Repertoire» Hypothesis: Repertoires of type three effectors are candidate determinants of host specificity in *Xanthomonas*. PLoS ONE.

[114] Collmer A., Badel J.L., Charkowski A.O., Deng W.-L., Fouts D.E., Ramos A.R., Rehm A.H., Anderson D.M., Schneewind O., Van Dijk K. (2000). *Pseudomonas syringae* Hrp type III secretion system and effector proteins. Proc. Natl. Acad. Sci. USA.

[115] Cunnac S., Lindeberg M., Collmer A. (2009). *Pseudomonas syringae* type III secretion system effectors: Repertoires in search of functions. Curr. Opin. Microbiol..

[116] Fouts D.E., Badel J.L., Ramos A.R., Rapp R.A., Collmer A. (2003). A *Pseudomonas syringae* pv. *tomato* DC3000 Hrp (type III secretion) deletion mutant expressing the Hrp system of bean pathogen *P. syringae* pv. *syringae* 61 retains normal host specificity for tomato. Mol. Plant Microbe Interact..

[117] Lindeberg M., Cunnac S., Collmer A. (2009). The evolution of *Pseudomonas syringae* host specificity and type III effector repertoires. Mol. Plant Pathol..

[118] Pensec F., Lebeau A., Daunay M.-C., Chiroleu F., Guidot A., Wicker E. (2015). Towards the identification of type III effectors associated with *Ralstonia solanacearum* virulence on tomato and eggplant. Phytopathology.

[119] Safni I., Cleenwerck I., De Vos P., Fegan M., Sly L., Kappler U. (2014). Polyphasic taxonomic revision of the *Ralstonia solanacearum* species complex: Proposal to emend the descriptions of *Ralstonia solanacearum* and *Ralstonia syzygii* and reclassify current *R. syzygii* strains as *Ralstonia syzygii* subsp. *syzygii* subsp. nov., *R. solanacearum* phylotype IV strains as *Ralstonia syzygii* subsp. *indonesiensis* subsp. nov., banana blood disease bacterium strains as *Ralstonia syzygii* subsp. *celebesensis* subsp. nov. and *R. solanacearum* phylotype I and III strains as *Ralstonia pseudosolanacearum* sp. nov. Int. J. Syst. Evol. Microbiol..

[120] Dalsing B.L., Truchon A.N., Gonzalez-Orta E.T., Milling A.S., Allen C. (2015). *Ralstonia solanacearum* uses inorganic nitrogen metabolism for virulence, ATP production, and detoxification in the oxygen-limited host xylem environment. mBio.

[121] Ailloud F., Lowe-Power T., Robène I., Cruveiller S., Allen C., Prior P. (2016). In planta comparative transcriptomics of host-adapted strains of *Ralstonia solanacearum*. PeerJ.

[122] Ailloud F., Lowe T., Cellier G., Roche D., Allen C., Prior P. (2015). Comparative genomic analysis of *Ralstonia solanacearum* reveals candidate genes for host specificity. BMC Genom..

[123] Zhang Y., Qiu S. (2015). Phylogenomic analysis of the genus *Ralstonia* based on 686 single-copy genes. Antonie van Leeuwenhoek.

[124] Castillo J.A., Agathos S.N. (2019). A genome-wide scan for genes under balancing selection in the plant pathogen *Ralstonia solanacearum*. BMC Evol. Biol..

[125] Cho H., Song E.-S., Heu S., Baek J., Lee Y.K., Lee S., Lee S.-W., Park D.S., Lee T.-H., Kim J.-G. (2019). Prediction of host-specific genes by pan-genome analyses of the Korean *Ralstonia solanacearum* species complex. Front. Microbiol..

[126] Perrier A., Barlet X., Rengel D., Prior P., Poussier S., Genin S., Guidot A. (2019). Spontaneous mutations in a regulatory gene induce phenotypic heterogeneity and adaptation of *Ralstonia solanacearum* to changing environments. Environ. Microbiol..

[127] Sabbagh C.R.R., Carrère S., Lonjon F., Vailleau F., Macho A.P., Genin S., Peeters N. (2019). Pangenomic type III effector database of the plant pathogenic *Ralstonia* spp.. PeerJ.

[128] Sang Y., Yu W., Zhuang H., Wei Y., Derevnina L., Yu G., Luo J., Macho A.P. (2020). Intra-strain elicitation and suppression of plant immunity by *Ralstonia solanacearum* type-III effectors in *Nicotiana benthamiana*. Plant Commun..

[129] Zheng X., Li X., Wang B., Cheng D., Li Y., Li W., Huang M., Tan X., Zhao G., Song B. (2019). A systematic screen of conserved *Ralstonia solanacearum* effectors reveals the role of RipAB, a nuclear-localized effector that suppresses immune responses in potato. Mol. Plant Pathol..

[130] Nakano M., Mukaihara T. (2019). Comprehensive identification of PTI suppressors in type III effector repertoire reveals that *Ralstonia solanacearum* activates jasmonate signaling at two different steps. Int. J. Mol. Sci..

[131] Chen B., Jiang H., Liao B., Ren X. (2007). Progress on groundnut genetic enhancement for bacterial wilt resistance. Chin. Agric. Sci. Bull..

[132] Deslandes L., Pileur F., Liaubet L., Camut S., Can C., Williams K., Holub E., Beynon J.L., Arlat M., Marco Y. (1998). Genetic characterization of RRS1, a recessive locus in *Arabidopsis thaliana* that confers resistance to the bacterial soilborne pathogen *Ralstonia solanacearum*. Mol. Plant Microbe Interact..

[133] Elsayed T.R., Jacquiod S., Nour E.H., Sørensen S.J., Smalla K. (2020). Biocontrol of bacterial wilt disease through complex interaction between tomato plant, antagonists, the indigenous rhizosphere microbiota, and *Ralstonia solanacearum*. Front. Microbiol..

[134] Fujiwara A., Fujisawa M., Hamasaki R., Kawasaki T., Fujie M., Yamada T. (2011). Biocontrol of *Ralstonia solanacearum* by treatment with lytic bacteriophages. Appl. Environ. Microbiol..

[135] Huet G. (2014). Breeding for resistances to *Ralstonia solanacearum*. Front. Plant Sci..

[136] Lemessa F., Zeller W. (2007). Screening rhizobacteria for biological control of *Ralstonia solanacearum* in Ethiopia. Biol. Control.

[137] Mansfield J.W. (2009). From bacterial avirulence genes to effector functions via the hrp delivery system: An overview of 25 years of progress in our understanding of plant innate immunity. Mol. Plant Pathol..

[138] Nguyen M., Ranamukhaarachchi S. (2010). Soil-borne antagonists for biological control of bacterial wilt disease caused by *Ralstonia solanacearum* in tomato and pepper. J. Plant Pathol..

[139] Wang J.-F., Ho F.-I., Truong H.T.H., Huang S.-M., Balatero C.H., Dittapongpitch V., Hidayati N. (2012). Identification of major QTLs associated with stable resistance of tomato cultivar ‘Hawaii 7996’ to *Ralstonia solanacearum*. Euphytica.

[140] Wei Z., Huang J., Yang T., Jousset A., Xu Y., Shen Q., Friman V.-P. (2017). Seasonal variation in the biocontrol efficiency of bacterial wilt is driven by temperature-mediated changes in bacterial competitive interactions. J. Appl. Ecol..

[141] Wei Z., Huang J.-F., Hu J., Gu Y.-A., Yang C.-L., Mei X.-L., Shen Q.-R., Xu Y., Friman V.-P. (2015). Altering transplantation time to avoid periods of high temperature can efficiently reduce bacterial wilt disease incidence with tomato. PLoS ONE.

[142] Wei Z., Yang X., Yin S., Shen Q., Ran W., Xu Y. (2011). Efficacy of *Bacillus*-fortified organic fertilizer in controlling bacterial wilt of tomato in the field. Appl. Soil Ecol..

[143] Yang L., Ding W., Xu Y., Wu D., Li S., Chen J., Guo B. (2016). New insights into the antibacterial activity of hydroxycoumarins against *Ralstonia solanacearum*. Molecules.

[144] Yuliar, Nion Y.A., Toyota K. (2015). Recent trends in control methods for bacterial wilt diseases caused by *Ralstonia solanacearum*. Microbes Environ..

[145] Zheng X., Zhu Y., Wang J., Wang Z., Liu B. (2019). Combined use of a microbial restoration substrate and avirulent *Ralstonia solanacearum* for the control of tomato bacterial wilt. Sci. Rep..

